# Gender Differences in Visuospatial Abilities and Complex Mathematical Problem Solving

**DOI:** 10.3389/fpsyg.2020.00191

**Published:** 2020-03-05

**Authors:** Isabel M. Ramírez-Uclés, Rafael Ramírez-Uclés

**Affiliations:** ^1^Department of Psychology of Personality, Evaluation and Psychological Treatments of the National University of Distance Education, Madrid, Spain; ^2^Department of Mathematics Education at the University of Granada, Granada, Spain

**Keywords:** mathematical talent, visual ability, gender differences, spatial tests, mathematical problem-solving

## Abstract

Mathematical problem-solving and spatial visualization are areas in which performance has been shown to vary with sex. This article describes the impact of gender on spatial relations measured in 331 secondary school students (202 males, 129 females), 145 (105 males, 40 females) of whom had been selected to participate in a mathematical talent stimulation project after passing a complex problem-solving test. In the two tests administered, the *Differential Aptitude Tests-Space Relations* (*DAT-SR*) and the *Primary Mental Abilities-Space Relations* (*PMA-SR*), performance was assessed on the grounds of both absolute scores and the ratio to the number of items answered. The students participating in the talent program earned higher scores on both tests, although no interaction was identified between mathematical abilities and gender in connection with the differences in spatial habilities observed. In PMA-SR, boys answered more items and scored higher, whereas in DAT-SR girls tended to omit more items. None of the indicators studied exhibited differences between the sexes in both tests and in some cases the differences in the absolute values of the indicators were absent when expressed as ratios.

## Introduction

Although the importance of visualization in mathematical problem solving has been highlighted in mathematics education (Clements and Battista, 1992; Arcavi, 2003), no consensus has yet been reached on its role in improving performance (Bishop, 1980; Lean and Clements, 1981). Traditional studies concluded that spatial awareness and the capacity to visualize abstract mathematical relationships were not necessarily components of mathematical talent (Krutetskii, 1976), whilst later studies revealed that talented students preferred non-visual methods (Presmeg, 1986). More recent research has found significant evidence of a relationship between visualization and mathematical ability, however (Rivera, 2011; Rabab’h and Veloo, 2015; Ramírez and Flores, 2017). The controversial findings are explained by the existence of different conceptions of mathematical talent and visualization, thereby requiring a clear view on what factors are used in the research to characterize both mathematical talent and visualization. Although there is consensus in that visualization should be considered an inherent ability needed to accomplish certain mathematical tasks, there is still no consensus on what instruments are most appropriate for identification of mathematical talent (Pitta-Pantazi and Christou, 2009).

A number of studies has focused on gender differences in these two areas, suggesting possible relationships between them (Ganley and Vasilyeva, 2011). Gender differences in spatial skills may serve as cognitive predictors of mathematical performance, particularly as regards geometry. Gender differences in spatial reasoning, together with the partial contribution of visual reasoning to problem solving, may have gender-related implications in mathematical contexts. Whilst males and females differ in spatial visualization and performance in high school geometry, however, their logical reasoning skills and use of geometric problem-solving strategies are indistinguishable (Battista, 1990).

This exploration of the effect of gender and mathematical performance on the differences observed in secondary school students’ visual abilities includes a review of the literature on gender differences in the two types of skills.

### Gender Differences in Mathematical Performance

Review papers and meta-analyses have identified greater mathematical problem-solving aptitudes among men (Maccoby and Jacklin, 1974; Hyde et al., 1990; Hyde, 2014). Hyde et al. (1990) reported wider differences between male and female secondary school students in complex problem solving than in parameters such as computation or understanding mathematical concepts. They observed no gender difference in arithmetic or algebra. Male superiority in geometry was minor, whilst the widest gender gap was recorded for tests with mixed content. The exercises used to assess mathematical performance have also been deemed to affect the results, with men performing better than women in problems involving mathematical reasoning (Halpern, 2000) and word problems which to be solved must be translated into mathematical terminology (Low and Over, 1993).

Other factors to be considered in gender difference studies is the date they are conducted and the group of people participating. A meta-analysis conducted 18 years later by Hyde et al. (2008) with 2nd to 11th year students in the United States revealed no difference between boys’ and girls’ lower level mathematical skills. When items entailing complex problem solving were included, girls in year 12 performed as well as their male classmates. Similarly, in a meta-analysis of studies conducted from 1990 to 2007, Lindberg et al. (2010) found only a minor difference between the sexes in complex problem solving. Else-Quest et al. (2010) conducted a meta-analysis of gender differences in mathematical performance, reporting substantial inter-country variability while also furnishing further evidence that, on average, males and females differ vary little in mathematics achievement, despite more positive attitudes toward mathematics among the former.

Although women continue to be underrepresented in science, technology, engineering and mathematics (STEM) education and careers (Else-Quest et al., 2013), gender differences in mathematical performance have been less consistently found (Ganley and Vasilyeva, 2011). Unlike other meta-analyses of performance in mathematical tests that reported males to perform more highly than females, a study on classroom gender differences authored by Voyer and Voyer (2014) found women to earn higher marks in all areas. That variability can be attributed to the diversity of the instruments used to measure mathematical performance (Gibbs, 2010). Boys have been perceived to be academically stronger in mathematics and science (Olszewski-Kubilius and Turner, 2002), with more male than female high-achievers in those subjects (Reis and Park, 2001). Gender differences have been recorded in tests assessing mathematical talent in students aged 12 to 14 (Benbow and Stanley, 1996). The decline in the male-female ratio among the highest scoring students in recent years calls for further study, however. Some authors have suggested that the male advantage in mathematical skills may be limited to the upper end of the talent distribution (Halpern et al., 2007).

Further to a meta-analysis of differences between the sexes in mathematics covering a number of countries (Else-Quest et al., 2010), the largest mean effect size was recorded in the PISA (*Program for International Student Assessment*) space/shape domain, which assesses the understanding of spatial relationships. The population studied, students aged 14 to 16, was deemed old enough to be able to solve complex mathematical problems. The data for that meta-analysis were drawn from the 2003 TIMSS (*Trends in International Mathematics and Science Study and the Program for International Student Assessment*), however, in which Spanish students did not participate. This study aims to further investigate on this particular group of students.

### Gender Differences in Visuospatial Ability: Performance Factors

Meta-analyses have consistently reported males to be more spatially skilled than females (Linn and Petersen, 1985; Hedges and Nowell, 1995; Voyer and Saunders, 2004; Halpern et al., 2007). A host of authors (Strand et al., 2006; Steinmayr and Spinath, 2008; Voyer and Voyer, 2014; Wach et al., 2015) has observed men to score higher than women on visuospatial tests, in particular in connection with mental rotation, where several authors observed a wider gap between men’s and women’s scores than in skills such as spatial perception or visualization (Voyer et al., 1995; Alansari et al., 2008; Geiser et al., 2008; Moè, 2009; Hyde, 2014; Xu et al., 2016). This study aims to look deeper into the prevalent role of mental rotation in gender differences, hence we will compare the results of a mental rotation test with those of another test related to the spatial ability of visualizing an object in three dimensions from a two-dimensional model.

Different performance factors have been identified in the effect of gender on mental rotation results, depending on the measuring instrument used and the conditions in which the tests were administered and scored. In a 3D mental rotation test measuring speed of performance as one such factor, time limits and the use of raw scores were found to benefit males (Goldstein et al., 1990). Loring-Meier and Halpern (1999) found males to answer more rapidly than females, whereas no difference was observed between them in the number of correct answers to items unrelated to mental rotation but involving visuospatial working memory. Robert and Chevrier (2003) reported similar numbers of correct answers among men and women when no time limit was established in mental rotation test, although men answered the items more quickly than women. Whilst some studies showed that such gender differences are more pronounced when the time to do the test is limited in mental rotation test (Voyer and Saunders, 2004; Peters, 2005; Voyer, 2011; Maeda and Yoon, 2016), others designed to assess mental rotation aptitudes reported no statistically significant differences between the sexes in completion time (Yoon and Mann, 2017). A third group observed males to score higher on visual tests irrespective of the existence of time limitations in mental rotation test (Delgado and Prieto, 1996; Geiser et al., 2006) or other figure analogy test (Blum et al., 2015). The use of ratios to score mental rotation performance significantly narrowed gender-related differences (Stumpf, 1993), whereas that approach reduced the gap between the two sexes’ scores for other aptitudes less meaningfully. Subsequent studies questioned the effect of these factors, confirming that the raw score-measured effect size of gender differences was unaffected when longer test times were allowed and that the reluctance to guess was similar for males and females, while males answered correctly to more exercises irrespective of timing (Delgado and Prieto, 1996). Masters (1998) found no evidence that the gender differences in mental rotation tests were affected by the scoring method or the time limit, with men scoring higher than women regardless of the scoring procedure. Other authors reported that the magnitude of gender differences in mental rotation was similar in distinct timing conditions when a conventional scoring method was used (Voyer et al., 2004).

The effect of time is associated with the strategy used to complete tests, with women being shown to be less self-assured when sitting these tests in mental rotation (Cooke-Simpson and Voyer, 2007) or in questionnaires about attitude and belief (Parsons et al., 1982) and as a result to adopt more conservative strategies in mental rotation test of other test (Hong and Aqui, 2004; Voyer and Saunders, 2004; Hirnstein et al., 2009). Research in figure analogy test has found women to be slower to answer and more reluctant to guess at answers in items they deem difficult, and hence to leave more items blank than males (Blum et al., 2015). A study of the impact of response latency, response frequency and time invested on a dynamic spatial test revealed that males outperformed females even when the effects of those performance factors were partial (Contreras et al., 2007). Other factors that may attenuate gender differences in mental rotation have also been identified, such as using the ratio of the correct to the attempted items as an alternative scoring criterion (Goldstein et al., 1990).

### Purpose of Study

The literature review conducted for this article revealed wider differences between the sexes in mental rotation than other spatial exercises. No consensus was detected, however, on how such differences may be impacted by scoring criteria, i.e., by the use of absolute values or the ratio of each to the number of items answered. The review also identified the early years of secondary school as the time when gender differences appear in complex mathematical problem solving. No conclusive evidence was found of interaction between spatial skills and complex problem-solving abilities in the differences between the sexes observed, particularly among Spanish students.

With a view to contributing to this issue, the research questions posed in this study were: do gender and the ability to solve complex problems affect the differences observed in the participants of the current study’ spatial aptitudes? If so, what performance measurements reflect that effect? To this end, results of 13- to 16-year old Spanish students are compared in two different test assessing the spatial ability (mental rotation and visualization of an object in three dimensions from a two-dimensional model) as well as the factors related to performance, completion time, and strategies used to answer the items.

## Materials and Methods

### Subjects

A total of 331 s, 2nd, 3rd and 4th -year secondary education students participated in this study. The mean age of the sample was 15 (±0.97) and the range 13 to 16. Part of the sample, 105 males and 40 females from nine provinces in Spain, were selected to participate in ESTALMAT, a project to encourage mathematically talented students, selected on the grounds of a math test in which the problems were divided into sections by level of difficulty. The participants didn’t receive any incentives. The test assessed students’ aptitude for and attitudes around mathematical knowledge. The differences in the number of boys and girls in this group attested to the differences between the sexes in complex problem-solving reported for youths of those ages, especially where the questionnaires combined areas such as geometry, arithmetic and logical reasoning (Hyde et al., 1990; Hyde, 2014). These students (‘’) had proven their ability to solve complex mathematical problems by passing a test with problems such as the following.

‘The vertices of a triangle bear the number 1 or −1 and the product of the three is shown in the middle. If we add the four numbers: (a) What values may the sum take? What combination yields zero? (b) What would the sum be if instead of a triangle we had a square? (c) If we use a polygon with an even number of sides, can the sum be zero? Why? (d) What sort of polygons with an odd number of sides could give us zero? Why?’

The 186 students (97 males and 89 females) in the other group were enrolled in 2nd, 3rd, or 4th-year secondary education in two schools, each in a different Spanish province. According to their teachers, these students (‘NCPs’) had exhibited no complex problem-solving talent.

With a view to exploring the issue in greater depth, this study analyzed the effect of gender and mathematical ability on performance in two spatial tests frequently used to diagnose spatial aptitudes in Spain.

### Materials

The following instruments were used in this study:

- *The Primary Mental Abilities Test (PMA)* – *Spatial Relations (SR)* (Thurstone and Thurstone, 1976). Thurstone’s initial battery of PMA tests yielded seven ‘primary mental abilities’: verbal comprehension (V), spatial orientation (S), inductive reasoning (I or R), number (N), word fluency (W), associative memory (M), and perceptual speed (P). The Spanish adaptation was created by TEA Ediciones in 1987. This study applied the test for spatial relations, defined in the Spanish edition as ‘the ability to interpret and recognize objects that change their spatial position, while maintaining their internal structure’. Cronbach’s alpha (a measure of reliability or internal consistency) for the SR factor has been shown to be 0.93, whilst the value calculated for the present sample was 0.89.

PMA-SR measures the ability to mentally rotate two-dimensional figures quickly and accurately (Linn and Petersen, 1985; Voyer, 2011). One of its features favored by researchers is the correction for guessing, for the final score is the number of correct minus the number of incorrect answers (Voyer and Saunders, 2004). Another prominent characteristic is the short time allowed, just 5 min, to answer 20 multiple-choice items, each with six options. Subjects consequently have an average of 15 s to analyze the six options in each item, without knowing how many are correct. Differences between the sexes have been identified for PMA-SR, with men scoring higher (Stericker and LeVesconte, 1982; Kail et al., 1984; Campos, 2014).

- *The Differential Aptitude Test (DAT-5) – Space Relations (SR)* (Bennett et al., 2000). The tests in the fifth version of the DAT assess eight aptitudes: verbal, numerical and abstract reasoning, perceptual speed and accuracy, mechanical reasoning, space relations and spelling and language usage. The Spanish adaptation of the original version was created in 2000 by TEA Ediciones. Level 1 of the space relations (SR) scale was chosen in this study to measure the ability to visualize an object in three dimensions from a two-dimensional model and mentally rotate the object in space. Cronbach’s alpha for groups participating in SR test Level 1 range from 0.86 to 0.93, whilst the value calculated for the present sample was 0.97.

Each test item consists in a two-dimensional drawing, which subjects must match to only one of four three-dimensional figures. This test is often used to study gender differences (Hartlage, 1970; Feingold, 1988; Delgado and Prieto, 1996), which have been identified by some authors (Hall, 1979) and reported by others to be minor only and less accentuated than observed with the mental rotation test (Linn and Petersen, 1985; Voyer et al., 1995; Kaufman, 2007). In this test subjects are given 20 min to choose one of four possible replies to each of 50 items. They must consequently answer each item in an average 24 s, although not all four choices must necessarily be analyzed, for participants know only one is correct.

Hereafter, the two aforementioned tests are referred to as PMA-SR and DAT-SR. The working hypothesis defined to explore the impact of gender differences and mathematical abilities on performance indicators was based on the earlier findings described above. The PMA-SR test was therefore deemed more appropriate to detect gender differences in spatial ability, for it measures mental rotation in a specific plane, whereas the DAT-SR test measures the ability to construct a three-dimensional object from its two-dimensional representation. The PMA-SR test might better identify gender differences in speed-related factors, given the short time afforded subjects to complete the exercise. The DAT-SR test, in turn, might furnish a more reliable measure of strategy-based self-confidence. Since there is only one correct answer to each item in DAT-SR, items left blank are a more sensitive indication of student uncertainty and therefore their level of self-confidence. More self-confident subjects would not need to analyze all the options as intensely and could consequently answer more quickly without leaving items blank.

### Procedure

The tests were administered to the original recommendations on instructions and timing. The talented complex problem-solvers sat the tests during one of their ESTALMAT project sessions, routinely conducted outside class time (on Saturday mornings). The PMA-SR instructions were delivered in 5 min, after which students were allowed 5 min to complete the test. After a 30 min break, the DAT-SR test was administered, again with a 5 min explanation followed in this case by 20 min to do the exercise. The same procedure was deployed with the control group students, who participated during normal classroom time.

As students were given no prior information about the scoring procedure, they did not know that the total score in PMA-SR was found as the difference between the number of correct and incorrect answers and in DAT-SR as the number of correct responses. They were, however, told that the number of correct choices per item in PMA-SR was indeterminate and that there was only one per item in DAT-SR.

All the subjects gave their consent to voluntarily participate in the study, which are compliant with the guidelines given by the Bioethics Committee from both UNED and University of Granada in relation to human subjects.

### Design and Variables

A 2 × 2, bi-factorial intergroup design was used, in which Gender (categories: male and female) and Ability (categories: CP, talented complex problem-solvers; and NCP, no complex problem-solving talent) were the independent variables. The dependent variables were performance, speed and confidence, measured in terms of the following indicators.

• Number of correct items (A1): in PMA-SR an item was deemed correct only if, of the six options given, all the actual rotations and no others were chosen. In DAT-SR an item was deemed correctly answered if the single correct option was chosen.
• Number of incorrect items (A2): in PMA-SR an item was deemed incorrect if any actual rotation was not chosen, or any non-rotations were. In DAT-SR, items were deemed incorrect when the wrong option was chosen.
• Number of items attempted (B1): the number of items attempted was the number answered: B1 = A1 + A2.
• Number of blank items (C1): blank items were all the ones where students chose none of the options. In PMA-SR, B1 + C1 = 20 and in DAT-SR, B1 + C1 = 50.
• Test score (A3): in PMA-SR the score was found by subtracting the number of incorrect from the number of correct items. In DAT-SR the score was the number of correctly answered items.
• Last item answered (B2): as the items were sorted correlatively, the value was the item answered that was numbered highest.
• Number of omissions (C2): the number of omissions was the number of items left blank prior to the last item answered. For PMA-SR, C2 + (20-B2) = C1 and for DAT-SR C2 + (50-B2) = C1.

Performance is measured by A3 indicator, which in DAT coincides with A1 whereas in PMA it also involves A2 for its calculation. B1 and B2 are speed indicators. C2 and C1 are used for measuring confidence, as they can differentiate whether an item is blank because of doubts in the correct answer or because of lack of time to answer it. The ratios of the number of correct answers and the number of items omitted to the number of items answered were used to infer the effectiveness of the strategy deployed (Goldstein et al., 1990; Delgado and Prieto, 1996):

• Number of correct answers/number of items answered (AR1).
• Number of items omitted/number of items answered (CR2).

### Data Analysis

In order to perform statistical analyses of data, those subjects whose protocols were incomplete or showed errors were removed from the analysis. First, the mean and standard deviation in the different scores was calculated (see Table 1), and the Kolmogorov–Smirnov test was used to assess the distribution of the scores. Determining the potential differences between groups in all variables was achieved through bifactorial intergroup 2 × 2 ANOVAs taking Gender and Ability as independent variables, and the scores obtained in PMA-SR and DAT-SR (absolute and ratio values) as dependent variables. Effect size was measured as partial eta-squared ($\eta_{\text{p}}^{2}$) and statistical significance was set at a confidence interval of 95%, with *p* < 0.05 as the accepted level of significance. All the analyses were performed using SPSS v.19 for Windows.

**TABLE 1:** Mean, standard deviation, and *F*-values for the parameters describing dependent variables mathematical talent and gender, expressed as absolute values: PMA-SR and DAT-SR tests.

	**Talented complex problem-solvers**				**Untalented**						
	**Male**		**Female**		**Male**		**Female**		***F*(1,323)**		
	***M***	***SD***	***M***	***SD***	***M***	***SD***	***M***	***SD***	**Ability**	**Gender**	**Interaction**
**Absolute values**					**PMA-SR**						

Right (A1)	11.40	3.85	10.83	3.67	7.73	4.52	5.95	4.01	77.60** (*p* = 0.000)	5.86* (*p* = 0.016)	1.54 (*p* = 0.216)
Wrong (A2)	2.22	1.91	1.88	1.56	4.61	4.22	4.88	4.02	46.60** (*p* = 0.000)	0.012 (*p* = 0.911)	0.594 (*p* = 0.911)
Score (A3)	32.26	10.92	30.55	10.06	23.40	12.57	18.49	11.63	58.41** (*p* = 0.000)	5.84* (*p* = 0.016)	1.36 (*p* = 0.244)
Attempted (B1)	13.62	3.83	12.70	3.66	12.34	3.94	10.82	3.57	12.29** (*p* = 0.000)	7.36** (*p* = 0.007)	0.436 (*p* = 0.510)
Last item (B2)	13.96	3.85	13.53	3.95	12.83	4.07	11.34	3.86	12.55** (*p* = 0.000)	4.26* (*p* = 0.040)	1.28 (*p* = 0.259)
Blank (C1)	6.38	3.83	7.30	3.66	7.66	3.94	9.17	3.57	12.29** (*p* = 0.001)	7.36** (*p* = 0.007)	0.436 (*p* = 0.517)
Omitted (C2)	0.34	1.06	0.83	2.37	0.49	1.94	0.51	1.69	0.163 (*p* = 0.687)	1.56 (*p* = 0.212)	1.30 (*p* = 0.253)

					**DAT-SR**						

Right (A1)	43.98	7.73	44.38	7.92	32.39	11.16	30.77	9.66	127.47** (*p* = 0.000)	0.299 (*p* = 0.585)	0.811 (*p* = 0.369)
Wrong (A2)	4.33	5.84	2.55	2.55	13.73	10.72	14.11	8.87	116.95** (*p* = 0.000)	0.515 (*p* = 0.474)	1.24 (*p* = 0.265)
Score (A3)	43.98	7.73	44.38	7.92	32.39	11.15	30.77	9.61	127.47** (*p* = 0.000)	0.299 (*p* = 0.585)	0.811 (*p* = 0.369)
Attempted (B1)	48.31	4.85	46.92	7.06	1.00	6.34	44.88	6.78	8.42** (*p* = 0.004)	3.20 (*p* = 0.074)	0.011 (*p* = 0.916)
Last item (B2)	48.57	4.37	47.95	5.04	46.40	6.16	45.85	6.70	10.04** (*p* = 0.002)	0.744 (*p* = 0.389)	0.003 (*p* = 0.957)
Blank (C1)	1.69	4.85	3.08	7.06	3.89	6.34	5.11	6.78	8.42** (*p* = 0.004)	3.20 (*p* = 0.074)	0.011 (*p* = 0.916)
Omitted (C2)	0.26	1.00	1.03	5.21	0.28	0.91	0.97	2.44	0.005 (*p* = 0.946)	6.85** (*p* = 0.009)	0.021 (*p* = 0.884)

**p < 0.01; *p < 0.05. In the table the exact values of *p* are presented for each value of *F*.

**TABLE 2:** Mean, standard deviations, and *F*-values for the parameters describing dependent variables mathematical talent and gender, expressed as the ratio to the number of items answered: PMA-SR and DAT-SR.

	**Talented complex problem-solvers**				**Untalented**						
	**Male**		**Female**		**Male**		**Female**		***F*(1,323)**		
	***M***	***SD***	***M***	***SD***	***M***	***SD***	***M***	***SD***	**Math**	**Gender**	**Interaction**
**Ratios**					**PMA-SR**						

Right (AR1)	0.83	0.13	0.84	0.14	0.62	0.29	0.54	0.30	78.61** (*p* = 0.000)	1.50 (*p* = 0.222)	0.250 (*p* = 0.114)
Omitted (CR2)	0.03	0.09	0.09	0.30	0.07	0.41	0.05	0.19	0.010 (*p* = 0.929)	0.456 (*p* = 0.500)	1.25 (*p* = 0.263)

					**DAT-SR**						

Right (AR1)	0.91	0.12	0.93	0.07	0.70	0.22	0.68	0.18	128.24** (*p* = 0.000)	0.129 (*p* = 0.720)	1.46 (*p* = 0.228)
Omitted (CR2)	0.007	0.04	0.05	0.31	0.007	0.02	0.02	0.06	1.16 (*p* = 0.282)	5.11* (*p* = 0.024)	1.11 (*p* = 0.291)

**p < 0.01; *p < 0.05. In the table the exact values of *p* are presented for each value of *F*.

## Results

### Absolute Values

CPs scored significantly higher than NCPs in all the performance indicators in both tests: more correct answers (A1) [*F*(1,323) = 77.60, *p* = 0.000, $\eta_{\text{p}}^{2}$ = 0.194 in PMA-SR; *F*(1,323) = 127.47, *p* = 0.000, $\eta_{\text{p}}^{2}$ = 0.283 in DAT-SR]; fewer incorrect answers (A2) [*F*(1,323) = 46.60, *p* = 0.000, $\eta_{\text{p}}^{2}$ = 0.126 in PMA-SR; *F*(1,323) = 116.95, *p* = 0.000, $\eta_{\text{p}}^{2}$ = 0.226 in DAT-SR]; and a higher score (A3) [*F*(1,323) = 58.41, *p* = 0.000, $\eta_{\text{p}}^{2}$ = 0.153 in PMA-SR; *F*(1,323) = 127.47, *p* = 0.000, $\eta_{\text{p}}^{2}$ = 0.283 in DAT-SR].

Gender had a significant effect on two of the performance indicators in PMA-SR, with males answering more items correctly (A1) [*F*(1,323) = 5.86, *p* = 0.016, $\eta_{\text{p}}^{2}$ = 0.016] and scoring higher [*F*(1,323) = 5.84, *p* = 0.016, $\eta_{\text{p}}^{2}$ = 0.018]. The differences in the number of incorrect responses (A2) were not statistically significant, however. Gender was not observed to prominently affect any of the performance indicators in DAT-SR. Nor was any significant interaction between the independent variables identified in any of the performance indicators in either test.

The CPs scored consistently higher in the speed indicators than the NCPs: more items attempted (B1) [*F*(1,323) = 12.29, *p* = 0.001, $\eta_{\text{p}}^{2}$ = 0.037 in PMA-SR; *F*(1,323) = 8.42, *p* = 0.004, $\eta_{\text{p}}^{2}$ = 0.025 in DAT-SR] and a larger number of last items answered (B2) [*F*(1,323) = 12.55, *p* = 0.000, $\eta_{\text{p}}^{2}$ = 0.037 in PMA-SR; *F*(1,323) = 10.04, *p* = 0.002, $\eta_{\text{p}}^{2}$ = 0.030 in DAT-SR].

In the PMA-SR test male subjects earned higher speed indicator scores, answered more items (B1) [*F*(1,323) = 7.36, *p* = 0.007, $\eta_{\text{p}}^{2}$ = 0.022] and completed more of the test by number of items answered (B2) than females [*F*(1,323) = 4.26, *p* = 0.040, $\eta_{\text{p}}^{2}$ = 0.013]. In contrast, gender had no significant effect on the DAT-SR test speed indicators, nor was any inter-variable interaction observed for speed in either of the two tests.

Problem-solving capacity exerted no prominent effect on the number of items omitted (C2) in either test, although talented complex problem-solvers left significantly fewer items blank (C1) [*F*(1,323) = 12.29, *p* = 0.001, $\eta_{\text{p}}^{2}$ = 0.037 in PMA-SR; *F*(1,323) = 8.42, *p* = 0.004, $\eta_{\text{p}}^{2}$ = 0.025 in DAT-SR].

Although no differences were observed between the sexes in the total number of items left blank in the DAT-SR test, obvious differences were recorded in the number omitted (C2) [*F*(1,323) = 6.85, *p* = 0.009, $\eta_{\text{p}}^{2}$ = 0.021].

The gender differences in the number of speed-related blank items found in PMA-SR were not observed in connection with omissions. In this test the mean number of omissions was less than half an item, an indication that subjects only exceptionally failed to answer due to uncertainty. As in the other indicators, no inter-variable interaction was observed in omissions.

### Ratios

CPs exhibited significantly higher AR1 scores than NCPs in both tests, denoting a higher percentage of correct answers and fewer errors [*F*(1,323) = 78.61, *p* = 0.000, $\eta_{\text{p}}^{2}$ = 0.196 in PMA-SR; *F*(1,323) = 128.24, *p* = 0.000, $\eta_{\text{p}}^{2}$ = 0.284, in DAT-SR]. Only minor differences were observed between the two groups in the number of items omitted, however, confirming the effectiveness of the non-omission strategy.

Males’ statistically significant higher absolute performance in terms of number of correct answers, scores and number of items answered in the PMA-SR test was absent in the AR1 findings. In other words, the differences between the sexes in the fraction of correct answers relative to the number of items answered were not significant.

In DAT-SR, as in the case of the absolute values which showed no differences in performance by sex, the AR1 ratio revealed the absence of significance between males’ and females’ likelihood of responding correctly to the items answered. In contrast, a significantly higher ratio of items omitted to items answered was observed for females (CR2) [*F*(1,323) = 5.11, *p* = 0.024, $\eta_{\text{p}}^{2}$ = 0.016].

## Discussion

This study used two spatial tests, PMA-SR and DAT-SR, to analyze the effect of gender and the ability to solve complex mathematical problems on performance. Gender (male/female) and mathematical ability (complex problem solvers/non-solvers) were the independent variables, while the performance indicators were score, number of correct and incorrect answers, number of items attempted, number left blank, number omitted and the last item answered, along with the ratios of the number of correct answers and the number of omissions to the total number of items answered. The study’s four major contributions to the effect of gender and mathematical talent on spatial aptitudes are highlighted below.

### Performance Was Higher Among Students With Complex Mathematical Problem-Solving Talent Than Among Their Less Talented Peers

CP students performed better and faster than NCPs in both tests administered here. The former were found to score significantly better than the latter in both tests: making fewer mistakes, leaving fewer items blank, answering more items, and exhibiting a higher success rate per item answered. The present findings therefore corroborate the positive relationship between mathematical talent and visual ability reported earlier (Rivera, 2011; Ramírez-Uclés et al., 2013; Rabab’h and Veloo, 2015; Ramírez and Flores, 2017), for the CP students in the sample implemented efficient test strategies, answering rapidly and omitting very few items.

### No Interaction Was Identified Between Ability to Solve Complex Problems and Gender

Although gender differences have been frequently and separately reported in studies of mathematical performance and visual skills, no interaction was observed in any of the indicators analyzed here. When explored together, the effect of one variable on the other was not determinant and the differences in mathematical ability were unrelated to the gender differences found in the tests. Nor did gender determine the differences observed in mathematical ability. Unlike other studies, the research conducted here was unable to confirm that differences between the sexes revealed by spatial tests concur with differences in complex problem-solving abilities (Olszewski-Kubilius and Turner, 2002). Nor was evidence found that such differences impact mathematical performance (Ganley and Vasilyeva, 2011). Although differences between the sexes in some indicators were apparently narrower in the CP group than in the sample as a whole, they were not statistically significant.

### None of the Indicators Denoted Significant Gender Differences in Both Tests

The inference drawn from the data, according to which none of the indicators denoted gender differences in both tests, is that the differences between the sexes in the performance factors were related to characteristics specific to each test. In other words, this study failed to find males more visually skilled, faster or more confident, for the differences in men’s and women’s scores were not observed consistently across the instruments and assessment criteria applied (Stumpf and Eliot, 1995; Gibbs, 2010). That boys scored significantly higher than girls in the PMA test while sex had no prominent effect of on the DAT test scores would seem to confirm that gender differences are better substantiated in mental rotation tests than in other spatial tests, as often described elsewhere (e.g., Voyer et al., 1995; Moè, 2009; Xu et al., 2016).

In this study, the performance differences observed in the PMA-SR test were speed-related, with males answering more items and completing more of the test, although at a success rate no higher than the females’ in any of the items. In this test, boys implemented a better strategy because it was faster, whereas they did not outperform the females in terms of success per item or number of omissions. Therefore, the strategy of answering more items per unit of time yields more correct responses per unit of time, as reported by other authors for mental rotation tests (Delgado and Prieto, 1996). The fact that only 9% of the subjects completed the PMA-SR test compared to 70% who completed the DAT-SR test attests to the need to answer more speedily to complete the former.

No differences between the sexes were observed in the speed or effectiveness indicators for DAT-SR. Differences were observed in that test with respect to omissions, with females more willing to leave an item blank when they were unsure of the answer. That finding was not consistent with results reported for an abridged version of the DAT-SR test, which revealed significant gender differences in the number of correct answers and items answered, but not in the absolute number of omissions or the ratio of omissions to the items answered (Delgado and Prieto, 1996). The characteristics of the two studies differed, however. Firstly, the earlier authors used an abridged version of DAT-SR (30 items) that was administered to two groups, one of which was allowed 12 and the other 25 min to complete the test. As that difference in timing spawned significant differences in the success rates relative to the items attempted, the effectiveness of the test was conditioned by that parameter. Secondly, in the present study the CPs performed better and faster, confirming that they differed significantly from the NCPs in respect of their mathematical skills. Similarly, 70% of the subjects in this research completed the full version of the DAT-SR (50 items in 20 min), compared to only 27.2% of the students in the earlier study who were given the same amount of time in items per minute.

Gender-related differences in strategy implemented varied depending on the test. In the PMA boys deployed faster strategies, whereas in the DAT test girls proved more reluctant to guess.

### Differences Between Absolute Variables and Ratios

The findings for the CP group were the same whether expressed as the absolute value of the variables or the value relative to the number of items attempted. The absolute DAT test results were likewise unchanged in any of the indicators when ratioed to the number of items attempted. In PMA-SR in contrast, the differences observed between the sexes in the absolute number of correct answers were absent when expressed as a fraction of the number of items answered, as observed by earlier authors (Goldstein et al., 1990; Stumpf, 1993). The strategy indicator ‘number of omissions’ yielded the same results in absolute and relative terms, a finding also consistent with other reports (Delgado and Prieto, 1996). In light of such disparity, the use of variable ratios cannot be said to necessarily narrow the gender gap observed.

### Implications and Limitations

Two limitations to this study are sample size and the smaller proportion of women. In relation to the sample, the results obtained are specific to the Spanish students who participated in the study, using the ability to solve complex mathematical problems as an indicator of mathematical ability, and the results obtained in PMA and DAT test as an indicator of spatial ability. Further generalization of the results of this study about gender differences in mathematical performance and visualization should take this limitation into account, as well as the heterogeneity of students with mathematical talent (Pitta-Pantazi and Christou, 2009). Another limitation stems from the smaller proportion of women in the sample selected, derived from their lower presence in the group of students selected to solve complex mathematical problems. Again, the results of this study should be interpreted under this limitation, which can itself be considered an indicator of sexes differences as found in certain contexts about mathematical abilities (Hyde et al., 1990; Hyde, 2014). In this sense, we consider that the assumption that females are not as capable in solving complex mathematical problems or spatial visualization tasks compared to males is wide-spread and, moreover, has often the character of a prejudice that may condition girls to not participate in some mathematical programs. It is necessary to investigate the specific factors that motivate these differences and not consider them as a “simple” effect of gender that may influence decisions in educational and social fields.

The inequalities between the CP and control groups were consistent with previous reports (Else-Quest et al., 2013; Hyde, 2014). This line of research would also benefit from a comparison to the results for other spatial tests and performance indicators. The present findings are nonetheless deemed to have significant implications, particularly for identifying gifted students or the direction adopted in future assessments of mathematical performance and visual ability. Affective factors associated with performance, speed or self-confidence have been shown to play different roles. In other words, the effect of greater self-confidence, greater speed or greater reluctance to guess on visual capacity might differ depending on the test. For instance, two subjects who work at different speeds might earn different scores in PMA-SR but the same in DAT-SR. By the same token, if one subject is more reluctant to guess than another, the two might earn the same scores in PMA-SR, but perform differently in DAT-SR. Just as the use of several instruments is recommended to identify gifted students (Pitta-Pantazi and Christou, 2009), the present authors believe a number of instruments should be deployed to assess visual ability and how they are impacted by other factors.

Although some of the test scores attest to differences between the sexes, an analysis of the cognitive aspects associated with such differences is believed to be in order. Despite the dependence of the reluctance to guess on personality factors, the parameter of greatest relevance may be the time invested in mentally rotating objects rather than the speed in answering or the decision to answer an item.

## Data Availability Statement

The datasets generated for this study are available on request to the corresponding author.

## Ethics Statement

The studies involving human participants were reviewed and approved by the Bioethics Committee of the University of Granada. Written informed consent to participate in this study was provided by the participants’ legal guardian/next of kin.

## Author Contributions

IR-U performed the overall planning and design of the study, the bibliographical revision, and the methodology and statistical analyses in the study. RR-U performed the assessment of the subjects, wrote the theoretical background, and derived the conclusions according to the results in the study.

## Conflict of Interest

The authors declare that the research was conducted in the absence of any commercial or financial relationships that could be construed as a potential conflict of interest.
